# Effect of silencing *Bemisia tabaci TLR3* and *TOB1* on fitness and begomovirus transmission

**DOI:** 10.3389/fpls.2023.1136262

**Published:** 2023-03-14

**Authors:** Pathukandathil Thesnim, Sumit Jangra, Manish Kumar, Amalendu Ghosh

**Affiliations:** ^1^ Insect Vector Laboratory, Advanced Centre for Plant Virology, ICAR-Indian Agricultural Research Institute, New Delhi, India; ^2^ Division of Entomology, ICAR-Indian Agricultural Research Institute, New Delhi, India

**Keywords:** whitefly, RNAi, ChiLCV, Toll-like receptor 3, transducer of erbB2.1, virus-vector relationship

## Abstract

*Bemisia tabaci* (Hemiptera: Aleyrodidae) is one of the most important invasive pests worldwide. It infests several vegetables, legumes, fiber, and ornamental crops. Besides causing direct damage by sucking plant sap, *B. tabaci* is the principal vector of begomoviruses. *Chilli leaf curl virus* (ChiLCV*, Begomovirus*) transmitted by *B. tabaci* is a major constraint in chilli production. *B. tabaci* genes associated with metabolism, signaling pathways, cellular processes, and organismal systems are highly enriched in response to ChiLCV infection. The previous transcriptome study suggested the association of *B. tabaci Toll-like receptor 3* (*TLR3*) and *transducer of erbB2.1* (*TOB1*) in ChiLCV infection. In the present study, *B. tabaci TLR3* and *TOB1* were silenced using double-stranded RNA (dsRNA) and the effect on fitness and begomovirus transmission has been reported. Oral delivery of dsRNA at 3 µg/mL reduced the expression of *B. tabaci TLR3* and *TOB1* by 6.77 and 3.01-fold, respectively. Silencing of *TLR3* and *TOB1* induced significant mortality in *B. tabaci* adults compared to untreated control. The ChiLCV copies in *B. tabaci* significantly reduced post-exposure to *TLR3* and *TOB1* dsRNAs. The ability of *B. tabaci* to transmit ChiLCV also declined post-silencing *TLR3* and *TOB1*. This is the first-ever report of silencing *B. tabaci TLR3* and *TOB1* to induce mortality and impair virus transmission ability in *B. tabaci*. *B. tabaci TLR3* and *TOB1* would be novel genetic targets to manage *B. tabaci* and restrict the spread of begomovirus.

## Introduction

Silverleaf whitefly [*Bemisia tabaci* (Gennadius), Hemiptera: Aleyrodidae] is a key agricultural pest of horticultural and field crops worldwide. Except for Antarctica, *B. tabaci* has been reported from all the continents. It infests over 900 plant species (Abd-Rabou and Simmons, 2010; Li et al., 2011). Adults and nymphs of *B. tabaci* cause economic damage to a wide range of vegetables, legumes, fibers, and ornamentals. *B. tabaci* causes direct damage to plants by sucking sap. Besides, it affects the quality and quantity of the produce by secreting honeydew and transmitting plant viruses (Jones, 2003; Hogenhout et al., 2008; Horowitz et al., 2020). *B. tabaci* transmits more than 400 plant viruses of the genera *Begomovirus* (*Geminiviridae*)*, Torradovirus* (*Secoviridae*), *Carlavirus* (*Betaflexiviridae*)*, Crinivirus* (*Colesteroviridae*), *Ipomovirus* (*Potyviridae*), *Polerovirus* (*Solemoviridae*), and *Cytorhabdovirus* (*Rhabdoviridae*) (Wang et al., 2020; Ghosh and Ghanim, 2021). *B. tabaci* is the principal vector of begomoviruses that cause annual losses of around 300 million US$ every year (Varma and Malathi, 2003; Navas-Castillo et al., 2011). The yield losses in vegetable crops caused by begomoviruses range from 20-100% (Polston and Anderson, 1997). *B. tabaci* transmits begomovirus in a persistent-circulative manner which means the virus particles need to cross the midgut barrier and reach the salivary glands through hemolymph in *B. tabaci* (Rosen et al., 2015; Czosnek et al., 2017). Several proteins such as heat shock protein 70 (hsp70), cyclophilin B (CypB), and peptidoglycan recognition protein (PGRP) were reported to interact with the coat protein of begomovirus in the midgut of *B. tabaci* (Götz et al., 2012; Kanakala and Ghanim, 2016; Wang et al., 2016). The current understanding of *B. tabaci*-begomovirus interactions is largely based on the tomato yellow leaf curl virus (TYLCV). Limited evidence is available on the functions of *B. tabaci* genes in the transmission of other predominant begomoviruses.

Chilli leaf curl virus (ChiLCV) poses a significant problem in chilli production especially in tropical and sub-tropical countries (Senanayake et al., 2007; Shingote et al., 2022). ChiLCV is a monopartite begomovirus with a circular, single-stranded DNA-A component of 2.7 kb and associated with alphasatellites (~1.4 kb) and/or betasatellites (~1.3 kb). The symptoms of ChiLCV include upward leaf curling, crinkling, puckering, and stunting of the entire plant. Under extreme circumstances, fruit yields have been recorded to drop by up to 100 percent (Thakur et al., 2018). The application of pesticides to curb *B. tabaci* is the primary control measure in practice (Horowitz et al., 2020). However, chemical insecticides are largely ineffective against *B. tabaci* due to the quick development of insecticide resistance. In India, an epidemic of *B. tabaci*-transmitted viral diseases during 2015-16 was mainly due to the failure of chemical pesticides (Verma and Bhattacharya, 2015; Kumar et al., 2020). Chemical insecticides also have the issue of health and environmental hazards.

Implementation of RNA interference (RNAi) would be a novel alternative for the sustainable management of *B. tabaci* and begomoviruses. RNAi is a eukaryotic gene silencing mechanism that has been employed to impair the development, fecundity, and survival of insect pests by knocking down key genes involved in the processes. However, inadequate information on the gene function of the target pest has restricted the scope of developing an RNAi-based pest management programme. Silencing of *B. tabaci hsp70* and *fasciclin 2* (*fas2)* modulates the ChiLCV transmission (Chakraborty and Ghosh, 2022). In our previous study, the differentially expressed genes (DEGs) of *B. tabaci* in response to ChiLCV have been reported (Nekkanti et al., 2022). DEGs associated with innate immunity such as *Toll-like receptor 3 (TLR3)*, *fas2*, *transducer of erbB2.1* (*TOB1*), and *GMP reductase* were highly abundant. Toll receptors induce interferons to confer antiviral resistance in vertebrates (Lester and Li, 2014). *TOB1* attenuates IRF3-directed antiviral responses by recruiting HDAC8 in virus-infected macrophages (Yu et al., 2022). However, the role of *TLR3* and *TOB1* in the virus transmission by *B. tabaci* or any other arthropods is not known. In the present study, *B. tabaci TLR3* and *TOB1* were chosen to be silenced through RNAi and the resultant effect on fitness and ChiLCV transmission ability of *B. tabaci* has been reported. To the best of our knowledge, this is the first evidence of functional validation of *B. tabaci TLR3* and *TOB1* for ChiLCV transmission.

## Materials and methods

### *B. tabaci* Asia II 1 population

An isofemale population of *B. tabaci* Asia II 1 being maintained at the whitefly rearing facility, Advanced Centre for Plant Virology, Indian Agricultural Research Institute (IARI), New Delhi since 2015 was used in the present study. The iso-female line was reared on eggplants, *Solanum melongena* (var. Navkiran, Mahyco, India) at 28 ± 2°C temperature, 60 ± 10% RH, and 16 hr light - 8 hr dark photoperiod. The identity of the pure culture was confirmed by sequencing of mitochondrial cytochrome oxidase subunit I (*mtCOI*).

### ChiLCV culture

The initial inoculum was taken from a pure culture of ChiLCV maintained at the laboratory by *B. tabaci-*inoculation. The culture was maintained on chilli plants (var. Priti, Nunhems) in insect-proof conditions. The identity of the virus was further confirmed by sequencing the DNA-A component amplified in PCR using primer pair, Begomo F-Begomo R (Akhter et al., 2009).

### Designing and synthesis of dsRNA

In our previous study, the expression of *B. tabaci TLR3* and *TOB1* was found highly abundant in response to ChiLCV infection (Nekkanti et al., 2022). In the present study, dsRNAs were designed to knock down *B. tabaci TLR3* and *TOB1*. The conserved regions were identified by aligning the sequences of *B. tabaci TLR3* and *TOB1* available in NCBI. Putative siRNAs in the conserved regions were identified using the siRNA Wizard online tool (https://www.invivogen.com/sirna-wizard, accessed on 12-12-2021). The regions with the maximum number of siRNAs were selected for designing dsRNA. The dsRNA stretch was further investigated for off-target effects with other organisms like humans, mice, birds, ants, and bees. A dsRNA targeting *Thrips palmi collagen alpha-1(III) chain-like* (*TpCOL3A1*) and not specific to *B. tabaci* was used as negative control.

The primer pairs were designed using the NCBI primer blast tool (https://www.ncbi.nlm.nih.gov/tools/primer-blast) to amplify the dsRNA stretches. The primers were validated and PCR conditions were optimized in a gradient PCR. The primer pairs used in the study are listed in **Table 1**. A 25 µL PCR mixture contained 1X PCR buffer (Thermo Fisher Scientific, USA), 0.4 µM of each forward and reverse primer (GCC Biotech, India), 0.26 mM dNTP mix (Thermo Fisher Scientific), 50 ng DNA template of *B. tabaci*, and 2 U of DreamTaq DNA polymerase (Thermo Fisher Scientific). PCR was performed in a T100 thermocycler (Bio-Rad, USA) with initial denaturation at 95°C for 5 min followed by 35 cycles of denaturation at 95°C for 40 s, annealing at 55°C for 40 s, extension at 72°C for 40 s, and a final extension at 72°C for 10 min. The amplified PCR products were resolved on 2% agarose gel along with 1 kb plus DNA ladder (Thermo Fisher Scientific) and visualized under a Gel documentation system (MaestroGen Inc, Taiwan).

**Table 1:** List of primers used in the study.

Sl. No.	Gene name	Primer Name	Primer Sequence (5´-3´)	Annealing temperature (°C) in PCR/qPCR	Amplicon size	Purpose	Reference
1	Begomovirus DNA-A	Begomo F	ACGCGTGCCGTGCTGCTGCCCCCATTGTCC	57	2.7 kb	Detection of begomovirus	Akhter et al., 2009
		Begomo R	ACGCGTATGGGCTGYCGAAGTTSAGAC				
2	ChiLCV coat protein	AG149F	TGAACAGGCCCATGAACAG	53	290 bp	Estimation of ChiLCV copies	Roy et al., 2021
		AG150R	ACGGACAAGGAAAAACATCAC				
3	*mtCOI* gene	C1-J-2195	TTGATTTTTTGGTCATCCAGAAGT	53	860 bp	Identification of cryptic species of *B. tabaci*	Simon et al., 1994
		L2-N-3014	TCCAATGCACTAATCTGCCATATTA				
4	*β-actin*	AG177F	ACATGGAAAAGATCTGGCAT	55	121 bp	Housekeeping gene	Chakraborty and Ghosh, 2022
		AG178R	TGAGTCATCTTTTCACGGTT				
5	*TOB1*	AG301F	AGGTCAGCTATAGGATTGGT	53	167 bp	dsRNA synthesis, qPCR	This study
		AG302R	TGAGCTGACTTAAACTGGAC				
6	*TLR3*	AG568F	GCATCGCAAAAGTATAAAGC	53	340 bp	dsRNA synthesis, qPCR	This study
		AG569R	CGAGACGTAGGAACTAATGT				

The amplified PCR products were eluted, ligated in the L4440 expression vector between two T7 promoters, and sequenced for further confirmation. The recombinant plasmids were transformed into RNase III mutant *E. coli* HT115 cells. The recombinant *E. coli* HT115 cells were induced with 0.8 M isopropyl-β-D-1-thiogalactopyranoside (IPTG) and cultured overnight at 37°C in a shaking incubator. Total RNA from the induced HT115 cells was extracted using Trizol reagent (Invitrogen, CA, USA) and resuspended in nuclease-free water. The dsRNA was purified by incubating with 1 U of RNase A, DNase and protease-free (Thermo Fisher Scientific) and 1 U of DNase I, RNase-free (Thermo Fisher Scientific) for 1 hr at 37°C in the presence of 500 mM sodium chloride as described by Chakraborty and Ghosh (2022). The enzymes were inactivated by chloroform extraction. The purified dsRNA was quantified in a spectrophotometer (NanoDrop 2000, Thermo Fisher Scientific), and visualized on 2% native agarose gel stained with GoodView (BR Biochem, India).

### Oral delivery of dsRNAs to *B. tabaci*

The purified *TLR3* and *TOB1* dsRNAs were separately delivered to *B. tabaci via* the oral feeding method described by Chakraborty and Ghosh (2022). Briefly, around 30 flies were collected in each cylindrical pet bottle (3.5 cm diameter, 16 cm height). The open end of the bottle was sealed with a stretched UV-sterilized Parafilm M. Based on our previous study, purified dsRNA at 3.0 μg/mL was supplemented with the artificial diet comprised of 20% sucrose and 5% yeast extract. The diet with dsRNA was sandwiched between two layers of stretched Parafilm M membrane. A diet without dsRNA and diet with *TpCOL3A1* dsRNA were served as control. For ventilation, a hole was made in the wall of the pet bottle and sealed with a muslin cloth. The pet bottles were kept in the upright position in dark at 26 ± 2°C and 60% RH. Three replicates were maintained for each treatment and repeated nine times. Percent mortality data was recorded 48 hr post dsRNA exposure. Tukey’s test was used to differentiate the mean differences across the categories with a 95% confidence interval using XLSTAT 2014.5.03. The surviving *B*. *tabaci* from these replicates were utilized to examine the relative expression of *TLR3* and *TOB1* mRNA and ChiLCV acquisition and transmission efficiency as described below.

### Estimating expression of *B. tabaci TLR3* and *TOB1*

The relative expression of *B. tabaci TLR3* and *TOB1* was estimated 48 hr post dsRNA feeding considering the *β-actin* gene as endogenous control. The primer pairs, AG301F-AG302R and AG568F-AG569R for *TOB1* and *TLR3*, respectively were used in the RT-qPCR assay (**Table 1**). Around 30 surviving *B. tabaci* in three replicates post *TLR3* and *TOB1* dsRNA exposure were used for total RNA isolation using Trizol reagent. The RNA was quantified in a spectrophotometer (NanoDrop 2000) and used for complementary DNA (cDNA) synthesis using the FIREScript RT cDNA synthesis kit (Solis BioDyne, Estonia). The 20 μL reaction mixture comprised of 1.0 μg RNA template, 5 μM oligo dT primers, 500 μM dNTP mix, 2 μL of 1 X reaction buffer, 10 U FIREScript RT, and 1 U RiboGrip RNase inhibitor. cDNA synthesis was carried out in a T100 thermocycler with reverse transcription at 42°C for 60 min, and enzyme inactivation at 85°C for 5 min. The relative RT-qPCR assay was performed in an Insta Q48M real-time PCR (Himedia, India). A 20 µL reaction mixture contained 1X GoTaq qPCR master mix (Promega, USA), 300 nM CXR passive reference dye, 0.25 µM of each forward and reverse primer, and 2 µL of template cDNA. Thermal cycling was performed at initial denaturation at 95°C for 5 min, 35 cycles of 95°C for 40 s, 55°C for 40 s, and 72°C for 40 s. After each reaction, a dissociation or melting curve was performed to evaluate the specificity of the amplicons. Three biological and two technical replicates were used in the RT-qPCR. The relative expression of mRNA in dsRNA-fed *B. tabaci* was measured in comparison to untreated control following the $2^{-\Delta \Delta \mathrm{CT}}$ method (Livak and Schmittgen, 2001). Microsoft Excel version 2016 was used to perform statistical analysis and prepare graphs. Expression of *B. tabaci TLR3* and *TOB1* in *TpCOL3A1* dsRNA-fed flies was considered as the negative control.

### Quantification of virus titer in *B. tabaci*

A portion of surviving flies post-dsRNA exposure was used to quantify the virus titer in *B. tabaci*. The flies were allowed to acquire ChiLCV by feeding on a ChiLCV-infected chilli plant (var. Preeti) for 24 hr. The ChiLCV copies acquired by dsRNA-exposed and nonexposed *B. tabaci* were estimated by absolute quantification in qPCR. DNA was isolated from the batch of 30 adult flies in three replicates using a CTAB extraction buffer as described by Roy et al. (2021) and quantified in a spectrophotometer. qPCR was performed in Insta Q48M real-time PCR with ChiLCV-specific primer pair, AG149F-AG150R (**Table 1**) (Roy et al., 2021). This was followed by a melting curve analysis to check the specificity of the reaction. Each treatment had three biological and two technical replicates. A standard curve of ChiLCV using primer pair AG149F-AG150R generated in our previous study (Chakraborty and Ghosh, 2022) was used to quantify the ChiLCV copies. The mean CT values obtained in qPCR were fitted into the standard curve and the resulting concentration was used for the calculation of virus copy number in Microsoft Excel 2016 using the following formula.


$$N=\left(x\times 6.022\times 10^{23}\right)/\left(n\times 660\times 10^{9}\right)$$

where N = number of viral copies, x = amount of amplicon in ng, and n = length of linearized plasmid DNA. The mean differences in virus copies were assessed for statistical significance by Tukey’s test at a confidence interval of 95% using XLSTAT 2014.5.03.

### Transmission of ChiLCV by *TLR3* and *TOB1* dsRNA-treated *B. tabaci*

To check the transmission efficacy of dsRNA-treated *B. tabaci*, a portion of *B. tabaci* exposed to ChiLCV for 24 hr was released onto the healthy chilli plants (var. Preeti) at the 3-4 leaf stage. They were allowed for 24 hr of inoculation feeding and eliminated manually. Ten plants in three replicates were used and four adult females per plant were released. The plants were maintained under insect-proof conditions and monitored for symptom development. *B. tabaci*, not exposed to dsRNA, were used as control. The ChiLCV infection in the inoculated plants was confirmed by ChiLCV-specific PCR at 21 days post-inoculation (dpi).

## Results

### *B. tabaci* population and ChiLCV culture

The identity of the *B. tabaci* population was confirmed by the nucleotide sequence of *mtCOI* gene. PCR with primer pair C1-J-2195 and L2-N-3014 amplified ~600 bp product as visualized on 1% agarose gel. The sequence analysis with BLASTn showed 100% homology to *B. tabaci* Asia II 1. The sequence submitted to GenBank can be retrieved by Accession No. OP223446.

PCR amplified a 2.7 kb product of full-length DNA-A segment from ChiLCV-infected plants. The sequence of DNA-A showed 100% homology to ChiLCV isolates upon BLASTn analysis. The sequence can be retrieved by Accession No. OM513903.

### Synthesis of dsRNA targeting *B. tabaci TLR3* and *TOB1*

Based on the multiple alignments of *B. tabaci TLR3* and *TOB1* sequences available in NCBI, the conserved 340 nt and 167 nt stretches of *TLR3* (~5.6 kb) and *TOB1* (~2.28 kb), respectively were chosen for dsRNA designing. The dsRNA sequences were unique to *B. tabaci* and no off-target hits were detected with *Homo sapiens* (taxid: 9605), Formicidae (taxid: 36668), mice (taxid: 10088), honeybees (taxid: 7460), and *Aves* (taxid: 8782) in blastn analysis.

**Figure 1:**
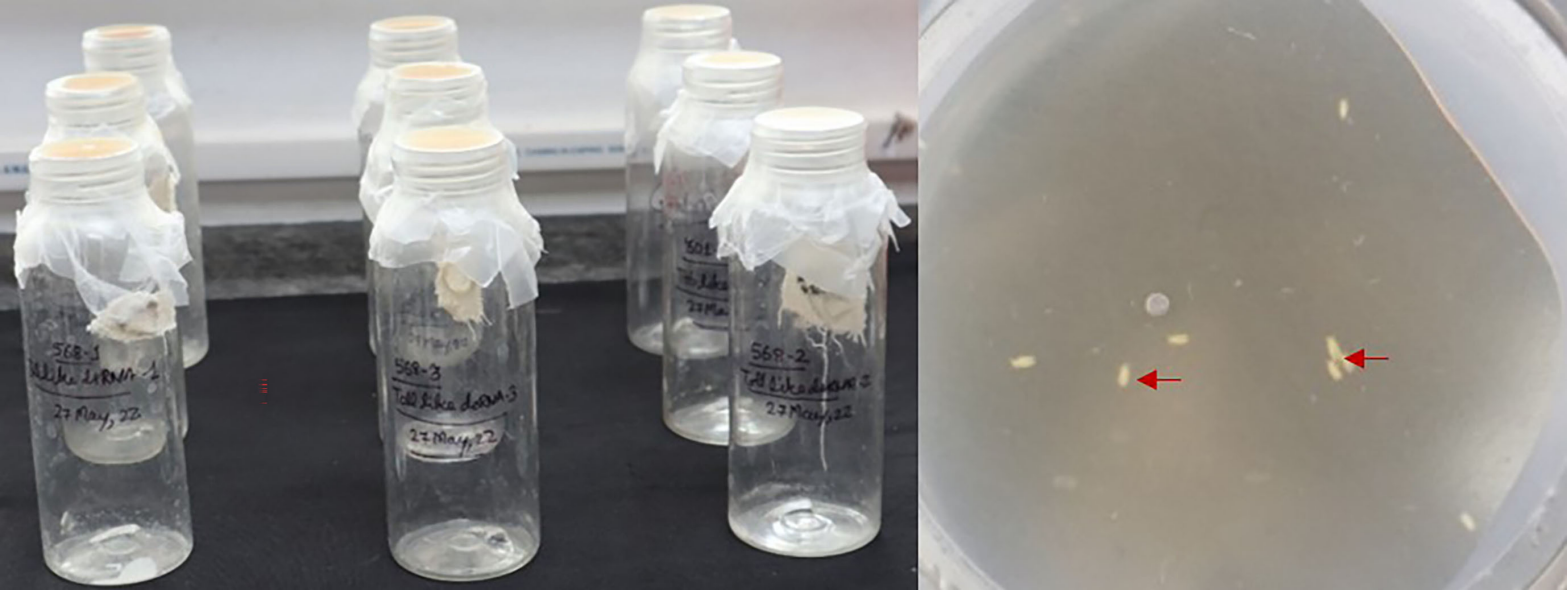
Delivery of dsRNA targeting *B. tabaci TOB1* and *TLR3*. Artificial feeding setup for *B. tabaci* adults. The artificial diet was supplemented with 3.0 μg/mL of dsRNA and sterile water (control). Red arrows show the *B. tabaci* adults feeding on the artificial diet.

PCR with primer pairs AG568F-AG569R and AG301F-AG302R produced amplicons of 340 bp and 167 bp for *B. tabaci TLR3* and *TOB1*, respectively (**Supplementary Figure 1**). The nucleotide sequences of the amplified products showed 100% homology with already available *B. tabaci TLR3* and *TOB1* sequences. The sequences can be retrieved by Accession No. OP784422 and OP219521. The dsRNA purified from total RNA using DNase I and RNase A produced single specific bands of ~340 bp and ~167 bp, respectively on 2% agarose gel (**Figure 1**). The concentration of *TLR3* dsRNA was 970.0 ng/µL, whereas it was 779.9 ng/µL in the case of *TOB1* dsRNA.

### Effect of *TLR3* and *TOB1* dsRNAs on target mRNA expression

**Figure 2:**
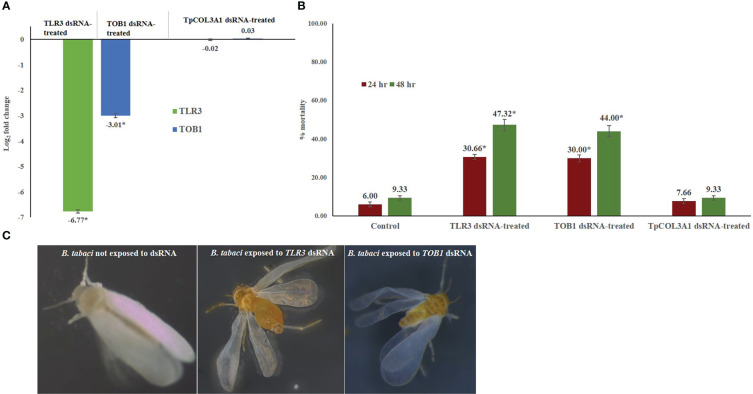
Effect of silencing *TOB1* and *TLR3* on survival of *B. tabaci*. **(A)** Normalized relative expression of *B. tabaci TOB1* and *TLR3* mRNA post 48 hr of dsRNA feeding. **(B)** Percent mortality of adult *B. tabaci* post *TLR3* and *TOB1* dsRNA feeding at 3.0 µg/mL. The mean denoted by an asterisk (*) indicates a significant difference (p< 0.0001). The error bars are the standard error of the mean (SEM). **(C)** *B. tabaci* adults fed on the diet mixed with *TLR3* and *TOB1* dsRNA and without dsRNA. No morphological deformities were observed in *TOB1* and *TLR3* dsRNA-fed *B. tabaci*.

The feeding of *TLR3* and *TOB1* dsRNAs significantly reduced the target gene expression in *B. tabaci* adults. In RT-qPCR analysis, the log $2^{-\Delta \Delta \mathrm{CT}}$ value of *B. tabaci TLR3* expression was 11.55 under normal conditions. Exposure to *TLR3* dsRNA significantly declined the *TLR3* mRNA level by 6.77-fold compared to the untreated control at 48 hr. The downregulation of target gene expression was consistent in all the biological replicates. Similarly, *TOB1* dsRNA significantly down-regulated the *TOB1* mRNA level by 3.01-fold (**Figure 2**). The log2^−ΔΔCT^ value of *B. tabaci TOB1* expression was 3.19 under normal conditions. The reduction in the target mRNA expression level of *B. tabaci* was significantly higher in *TLR3* dsRNA exposure than *TOB1* dsRNA at 48 hr after oral delivery. The expression of *B. tabaci TLR3* and *TOB1* post *TpCOL3A1* dsRNA exposure was statistically at par with untreated control. There was no significant regulation of the endogenous control gene, *β-actin* between dsRNA-exposed and non-exposed *B. tabaci* populations which indicated the specificity of the *TLR3* and *TOB1* dsRNAs on the target mRNAs. The melting curve analysis in RT-qPCR showed that the primer pairs for *TLR3*, *TOB1* and *β-actin* did not produce any secondary peaks that indicated the specificity of the reactions.

### Effect of silencing *B. tabaci TLR3* and *TOB1* on *B. tabaci* fitness

Feeding on *TLR3* and *TOB1* dsRNAs significantly altered the fitness of *B. tabaci* under controlled conditions (**Figure 2**). *TLR3* and *TOB1* dsRNA feeding at a concentration of 3.0 μg/mL induced mortality in *B. tabaci* adults. A mortality of 30.66% was recorded in *TLR3* dsRNA-fed *B. tabaci* 24 hr post-feeding, whereas it was 29.99% when fed on *TOB1* dsRNA. The mortality further increased with an increase in the exposure period. Up to 47.32% mortality was recorded 48 hr post-feeding on *TLR3* dsRNA. In the case of *TOB1* dsRNA, the mortality increased up to 43.99% compared to *B. tabaci* fed on a diet without dsRNA (9.33%). However, no morphological deformities were recorded in the *TLR3* or *TOB1* dsRNA-fed *B. tabaci* when observed under a microscope (**Figure 2**). Mortality induced by *TLR3* and *TOB1* dsRNAs was found to be significant at p<0.0001 at a confidence limit of 95%. There was no significant mortality of *B. tabaci* post *TpCOL3A1* dsRNA exposure compared to untreated control.

### Effect of *TLR3* and *TOB1* silencing on ChiLCV acquisition and transmission by *B. tabaci*

**Figure 3:**
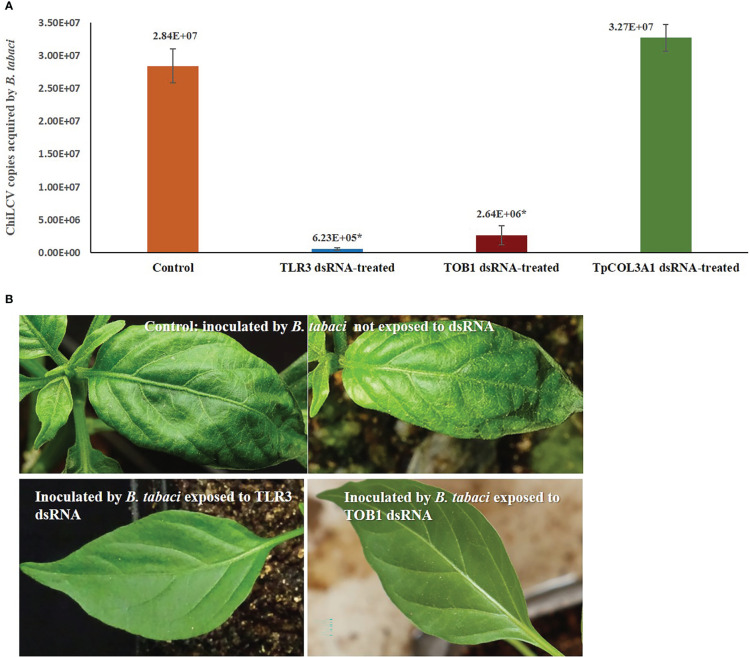
Effect of silencing *TLR3* and *TOB1* on ChiLCV acquisition and transmission by *B. tabaci*. **(A)** Mean ChiLCV copy numbers acquired by *TLR3* and *TOB1* dsRNA-fed *B. tabaci*. The mean denoted by an asterisk (*) indicates a significant difference (p< 0.0001). The error bars are the standard error of the mean (SEM). **(B)** Leaf curl symptoms on chilli plants inoculated by *B. tabaci* not exposed to dsRNA and exposed to *TLR3* and *TOB1* dsRNAs.

Silencing of *B. tabaci TLR3* and *TOB1* significantly decreased the ChiLCV titer within *B. tabaci.* The mean ChiLCV copy number was 2.84 x 10^7^ in *B. tabaci* fed on the diet without dsRNA (**Figure 3**). Exposure to *TLR3* dsRNA at 3 μg/mL induced 45.58 -fold reduction (6.23 x 10^5^ copies) in the mean ChiLCV copy. The decrease in ChiLCV copy in *TOB1* dsRNA-treated *B. tabaci* was comparatively lower than in *TLR3* dsRNA treatment. The ChiLCV titer in *B. tabaci* was reduced by 10.75-fold (2.64 x 10^6^ copies) post *TOB1* dsRNA exposure. There was no significant change in ChiLCV titer post *TpCOL3A1* dsRNA exposure compared to untreated control.

*B. tabaci* that were not exposed to *TLR3* and *TOB1* dsRNAs successfully transmitted ChiLCV to the inoculated plants. 93.33% of the inoculated plants tested positive in PCR with ChiLCV-specific primers. The infected plants showed characteristic ChiLCV symptoms like vein clearing, curling, and twisting of leaves, reduction of leaf size, puckering, reduction in inter-nodal length, thickening of leaves, swelling of veins, and overcrowding of leaves (**Figure 3**). Chilli plants inoculated by *B. tabaci* exposed to *TLR3* and *TOB1* dsRNAs showed no symptoms up to 21 dpi. No amplification specific to ChiLCV was recorded in PCR for the plants inoculated by dsRNA-fed *B. tabaci.*

## Discussion

Several transcripts of *B. tabaci* are differentially regulated upon ChiLCV infection, yet the functional roles of these genes in virus transmission remain unknown. In ChiLCV-infected *B. tabaci, TLR3* and *TOB1* were highly abundant in adult *B. tabaci* which might facilitate the invasion and multiplication of the virus in *B. tabaci* (Nekkanti et al., 2022). Toll-like receptors are actively involved in innate immune responses against viruses (Perales-Linares and Navas-Martín, 2013; Lester and Li, 2014; He et al., 2021) and *TLR3* is involved in protective antiviral responses (Ozato et al., 2002; Fernandes-Santos and Azeredo, 2022). Perales-Linares and Navas-Martín (2013) reported that *TLR3* participates in both defense and offense in host immunity to viruses. *TOB1* attenuates IRF3-directed antiviral responses by recruiting HDAC8 to specifically suppress IFN-β expression in virus-infected macrophages. *TOB1* deficiency enhanced antiviral response and suppressed viral replication *in vivo* (Yu et al., 2022). The present study aimed to understand the functional role of *B. tabaci TLR3* and *TOB1* in ChiLCV infection.

dsRNAs targeting *B. tabaci TLR3* and *TOB1* were orally administered to *B. tabaci* adults in the present study. The expression of *TLR3* and *TOB1* mRNA was down-regulated by 6.67- and 3.01-folds, respectively 48 hr post-dsRNA feeding. dsRNA targeting *T. palmi COL3A1* was taken as a negative control in *B. tabaci*. There was no significant regulation in the expression of *TL3* and *TOB1* post *TpCOL3A1* dsRNA exposure. Significant downregulation of target genes by oral delivery of dsRNA was previously reported for *B. tabaci ribosomal protein L9 (RPL9), vacuolar-type ATPase subunit A (V-ATPase A), cytochrome P450 family 3 subfamily A polypeptide 1 (Cyp315a1), Cyp18a1, ecdysone receptor gene (EcR5), ecdysone inducible gene (E75), hsp70*, and *fas2* (Upadhyay et al., 2011; Luan et al., 2013; Vyas et al., 2017; Chakraborty and Ghosh, 2022). Feeding on transgenic plants expressing dsRNA showed a down-regulation of around 90% in *B. tabaci aquaporin (AQP)* mRNA level 24 hr post-feeding (Raza et al., 2016). The difference in the level of silencing might be due to the variation in concentration of dsRNA, exposure period, target mRNA copies, delivery method, and the host’s defense mechanism (Ramkumar et al., 2021).

Silencing of *B. tabaci TLR3* and *TOB1* also significantly altered the survival of *B. tabaci* adults. Silencing of *B. tabaci TLR3* induced a mean mortality of 30.66% 24 hr post-dsRNA exposure which increased to 47.32% at 48 hr. Similarly, the silencing of *B. tabaci TOB1* caused up to 43.99% mortality compared to *B. tabaci* (9.33%) fed on a diet without dsRNA and diet with *TpCOL3A1* dsRNA. *B. tabaci TLR3* and *TOB1* are involved in a network of molecular and biological processes. Loss of *TLR3* and *TOB1* functions due to depletion of mRNA might hamper the critical physiological processes in *B. tabaci*, leading to mortality. Silencing of *actin ortholog*, *ADP/ATP translocase*, *α-tubulin*, *ribosomal protein L9 (RPL9)*, and *V-ATPase A subunit* also caused 27-97% mortality in *B. tabaci* (Upadhyay et al., 2011). Significant mortality was reported in *B. tabaci* upon silencing *AQP*, *calcitonin* (*CAL*), *SWItch/sucrose non-fermentable* (*SNF7*), *inhibitor of apoptosis* (*IAP*)*, hsp20, hsp40*, *knottin-1* (*k-1*), *CypB*, *hsp70*, and *fas2* (Kaur et al., 2020; Chakraborty and Ghosh, 2022). Morphological deformities like twisting wings were reported post-silencing *B. tabaci hsp70* (Kanakala et al., 2019). However, no such morphological abnormalities were recorded post-silencing *B. tabaci TLR3* and *TOB1* in the present study. Probably, *TLR3* and *TOB1* are not involved in any morphogenesis of *B. tabaci* or the exposure was too short to induce any morphological deformities.

Further, silencing of *B. tabaci TLR3* and *TOB1* reduced the ability of *B. tabaci* to acquire and transmit ChiLCV. The mean ChiLCV copies acquired by *B. tabaci* were decreased by 45.58 and 10.75- folds post-silencing *TLR3* and *TOB1* compared to *B. tabaci* fed on the diet without dsRNA and diet with *TpCOL3A1* dsRNA. There were no symptoms in chilli plants up to 21 dpi when inoculated by *B. tabaci* fed on a diet mixed with *TLR3* or *TOB1* dsRNA. In contrast to the expectation, the results indicated that *TLR3* is not involved in viral defense in *B. tabaci* as silencing of *TLR3* decreases ChiLCV titer and transmission ability. Whereas, a reduction in ChiLCV titer post-silencing *TOB1* supports its negative regulatory role in viral defense. Moreover, both target genes are essential for normal physiological functions in *B. tabaci*. Virus transmission might also be affected due to the poor physiological fitness of *B. tabaci* resulting from the depletion of *TLR3* and *TOB1* mRNAs. The results indicated that *B. tabaci TLR3* and *TOB1* would be novel targets to induce mortality and reduce ChiLCV transmission by *B. tabaci*. Complete inhibition of the ChiLCV transmission ability of *B. tabaci* was earlier demonstrated by the spray-on application of naked *hsp70* dsRNA under controlled conditions (Chakraborty and Ghosh, 2022). A similar strategy to restrict begomovirus spread by *B. tabaci* was used by Wang et al. (2016) by silencing *B. tabaci defensin-like* gene (*Btdef*) which led to a lower accumulation of tomato yellow leaf curl China virus in *B. tabaci*. However, the silencing of *fas2* increased the ChiLCV acquisition by *B. tabaci* (Chakraborty and Ghosh, 2022).

The present study is the first to demonstrate the involvement of *B. tabaci TLR3* and *TOB1* in the survival of *B. tabaci* and ChiLCV transmission by RNAi. It enriches our understanding of the gene functions of *B. tabaci* in begomovirus transmission. The outcome of the study would enable an in-depth study on the functional genomics of *B. tabaci* and apprise the *B. tabaci*-begomovirus relationships.

## Data availability statement

The original contributions presented in the study are included in the article/**Supplementary Material**. Further inquiries can be directed to the corresponding author.

## Author contributions

AG conceived and designed the research and wrote and edited the final manuscript. PT, SJ, and MK conducted the experiments, recorded the experimental data, and wrote the draft manuscript. AG and SJ reviewed the data. All authors read and approved the manuscript. All authors contributed to the article and approved the submitted version.

## References

[B1] Abd-Rabou S. Simmons A. M. (2010). Survey of reproductive host plants of *Bemisia tabaci* (Hemiptera: Aleyrodidae) in Egypt, including new host records. Entomol. News 121, 456–465. doi: 10.3157/021.121.0507

[B2] Akhter A. Qazi J. Saeed M. Mansoor S. (2009). A severe leaf curl disease on chilies in Pakistan is associated with multiple begomovirus components. Plant Dis. 93, 962. doi: 10.1094/PDIS-93-9-0962B 30754557

[B3] Chakraborty P. Ghosh A. (2022). Topical spray of dsRNA induces mortality and inhibits chilli leaf curl virus transmission by bemisia tabaci Asia II 1. Cells 11, 833. doi: 10.3390/cells11050833 35269455 PMC8909865

[B4] Czosnek H. Hariton-Shalev A. Sobol I. Gorovits R. Ghanim M. (2017). The incredible journey of begomoviruses in their whitefly vector. Viruses 9, 273. doi: 10.3390/v9100273 28946649 PMC5691625

[B5] Fernandes-Santos C. Azeredo E. L. (2022). Innate immune response to dengue virus: Toll-like receptors and antiviral response. Viruses 14, 992. doi: 10.3390/v14050992 35632732 PMC9147118

[B7] Ghosh S. Ghanim M. (2021). Factors determining transmission of persistent viruses by bemisia tabaci and emergence of new virus-vector relationships. Viruses 13, 1808. doi: 10.3390/v13091808 34578388 PMC8472762

[B8] Götz M. Popovski S. Kollenberg M. Gorovits R. Brown J. K. Cicero J. M.. (2012). Implication of bemisia tabaci heat shock protein 70 in begomovirus-whitefly interactions. J. Virol. 86, 13241–13252. doi: 10.1128/JVI.00880-12 23015709 PMC3503126

[B9] He Y. J. Lu G. Qi Y. H. Zhang Y. Zhang X. D. Huang H. J.. (2021). Activation of toll immune pathway in an insect vector induced by a plant virus. Front. Immunol. 11, 613957. doi: 10.3389/fimmu.2020.613957 33488623 PMC7821435

[B10] Hogenhout S. A. Ammar e.-D. Whitfield A. E. Redinbaugh M. G. (2008). Insect vector interactions with persistently transmitted viruses. Annu. Rev. Phytopathol. 46, 327–359. doi: 10.1146/annurev.phyto.022508.092135 18680428

[B11] Horowitz A. R. Ghanim M. Roditakis E. Nauen R. Ishaaya I. (2020). Insecticide resistance and its management in *Bemisia tabaci* species. J. Pest Sci. 93, 893–910. doi: 10.1007/s10340-020-01210-0

[B12] Jones D. R. (2003). Plant viruses transmitted by whiteflies. Eur. J. Plant Pathol. 109, 195–219. doi: 10.1023/A:1022846630513

[B13] Kanakala S. Ghanim M. (2016). Implication of the whitefly *Bemisia tabaci* cyclophilin b protein in the transmission of tomato yellow leaf curl virus. Front. Plant Sci. 7, 1702. doi: 10.3389/fpls.2016.01702 27895657 PMC5109225

[B14] Kanakala S. Kontsedalov S. Lebedev G. Ghanim M. (2019). Plant-mediated silencing of the whitefly bemisia tabaci cyclophilin b and heat shock protein 70 impairs insect development and virus transmission. Front. Physiol. 10, 557. doi: 10.3389/fphys.2019.00557 31133883 PMC6517521

[B15] Kaur R. Gupta M. Singh S. Joshi N. Sharma A. (2020). Enhancing RNAi efficiency to decipher the functional response of potential genes in *Bemisia tabaci* AsiaII-1 (Gennadius) through dsRNA feeding assays. Front. Physiol. 11, 123. doi: 10.3389/fphys.2020.00123 32194431 PMC7061899

[B16] Kumar V. Kular J. S. Kumar R. Sidhu S. S. Chhuneja P. K. (2020). Integrated whitefly [*Bemisia tabaci* (Gennadius)] management in bt-cotton in north India: An agroecosystem-wide community-based approach. Curr. Sci. 119 (4), 618–624. doi: 10.18520/cs/v119/i4/618-624

[B17] Lester S. N. Li K. (2014). Toll-like receptors in antiviral innate immunity. J. Mol. Biol. 426, 1246–1264. doi: 10.1016/j.jmb.2013.11.024 24316048 PMC3943763

[B18] Li S. J. Xue X. Ahmed M. Z. Ren S. X. Du Y. Z. Wu J. H.. (2011). Host plants and natural enemies of *Bemisia tabaci* (Hemiptera: Aleyrodidae) in China. Insect Sci. 18, 101–112. doi: 10.1111/j.1744-7917.2010.01395.x

[B19] Livak K. J. Schmittgen T. D. (2001). Analysis of relative gene expression data using real-time quantitative PCR and the 2^–ΔΔC^ _T_ method. Methods 25, 402–408. doi: 10.1006/meth.2001.1262 11846609

[B20] Luan J. B. Ghanim M. Liu S. S. Czosnek H. (2013). Silencing the ecdysone synthesis and signaling pathway genes disrupts nymphal development in the whitefly. Insect Biochem. Mol. Biol. 43, 740–746. doi: 10.1016/j.ibmb.2013.05.012 23748027

[B22] Navas-Castillo J. Fiallo-Olivé E. Sánchez-Campos S. (2011). Emerging virus diseases transmitted by whiteflies. Annu. Rev. Phytopathol. 49, 219–248. doi: 10.1146/annurev-phyto-072910-095235 21568700

[B23] Nekkanti A. Chakraborty P. Ghosh A. Iquebal M. A. Jaiswal S. Baranwal V. K. (2022). Transcriptomic changes of *Bemisia tabaci* Asia II 1 induced by chilli leaf curl virus trigger infection and circulation in its vector. Front. Microbiol. 13, 423–466. doi: 10.3389/fmicb.2022.890807 PMC9096263 35572639

[B24] Ozato K. Tsujimura H. Tamura T. (2002). Toll-like receptor signaling and regulation of cytokine gene expression in the immune system. BioTechniques Suppl. 66-8, 70, 72 passim. doi: 10.2144/Oct0208 12395929

[B25] Perales-Linares R. F. Navas-Martín S. (2013). Toll-like receptor 3 in viral pathogenesis: Friend or foe? Immunology 140, 153–167. doi: 10.1111/imm.12143 23909285 PMC3784162

[B26] Polston J. E. Anderson P. K. (1997). The emergence of whitefly-transmitted geminiviruses in tomato in the Western hemisphere. Plant Dis. 81, 1358–1369. doi: 10.1094/PDIS.1997.81.12.1358 30861786

[B27] Ramkumar G. Asokan R. Prasannakumar N. R. Kariyanna B. Karthi S. Alwahibi M. S.. (2021). RNA Interference suppression of v-ATPase b and juvenile hormone binding protein genes through topically applied dsRNA on tomato leaves: Developing biopesticides to control the south American pinworm, *Tuta absoluta* (Lepidoptera: Gelechiidae). Front. Physiol. 12, 742871. doi: 10.3389/fphys.2021.742871 34867448 PMC8637209

[B40] Raza A Malik H.J. Shafiq M. Amin I. Scheffler J.A. Scheffler B.E. Mansoor S. (2016). RNA interference based approach to down regulate osmoregulators of whitefly (*Bemisia tabaci*): potential technology for the control of whitefly. PLoS ONE 11(4), e0153883. doi: 10.1371/journal.pone.0153883 27105353 PMC4841547

[B28] Rosen R. Kanakala S. Kliot A. Cathrin Pakkianathan B. Farich B. A. Santana-Magal N.. (2015). Persistent, circulative transmission of begomoviruses by whitefly vectors. Curr. Opin. Virol. 15, 1–8. doi: 10.1016/j.coviro.2015.06.008 26196230

[B29] Roy B. Chakraborty P. Ghosh A. (2021). How many begomovirus copies are acquired and inoculated by its vector, whitefly (Bemisia tabaci) during feeding? PloS One 16, e0258933. doi: 10.1371/journal.pone.0258933 34699546 PMC8547624

[B30] Senanayake D. M. J. B. Mandal B. Lodha S. Varma A. (2007). First report of chilli leaf curl virus affecting chilli in India. Plant Pathol. 56, 343–343. doi: 10.1111/j.1365-3059.2007.01513.x

[B31] Shingote P. R. Wasule D. L. Parma V. S. Holkar S. K. Karkute S. G. Parlawar N. D.. (2022). An overview of chili leaf curl disease: Molecular mechanisms, impact, challenges, and disease management strategies in Indian subcontinent. Front. Microbiol. 29. doi: 10.3389/fmicb.2022.899512 PMC9277185 35847087

[B41] Simon C. Frati F. Beckenbach A.T. Crespi B.J. Liu H. Flook P.K. (1994). Evolution, weighting, and phylogenetic utility of mitochondrial gene sequences and a compilation of conserved polymerase chain reaction primers. Ann. Entomol. Soc. Am 87, 651–701. doi: 10.1093/AESA/87.6.651

[B32] Thakur H. Jindal S. K. Sharma A. Dhaliwal M. S. (2018). Chilli leaf curl virus disease: A serious threat for chilli cultivation. J. Plant Dis. Prot. 125, 239–249. doi: 10.1007/s41348-018-0146-8

[B33] Upadhyay S. K. Chandrashekar K. Thakur N. Verma P. C. Borgio J. F. Singh P. K.. (2011). RNA Interference for the control of whiteflies (*Bemisia tabaci*) by oral route. J. Biosci. 36, 153–161. doi: 10.1007/s12038-011-9009-1 21451256

[B34] Varma A. Malathi V. G. (2003). Emerging geminivirus problems: A serious threat to crop production. Ann. Appl. Biol. 142, 145–164. doi: 10.1111/j.1744-7348.2003.tb00240.x

[B35] Verma S. Bhattacharya A. (2015)Whitefly destroys 2/3rd of punjab’s cotton crop, 15 farmers commit suicide. In: Times of India. Available at: http://timesofindia.indiatimes.com/articleshow/49265083.cms?source=contentofinterest&utm_medium=text&utm_campaign=cpps (Accessed 21-02-2023).

[B36] Vyas M. Raza A. Ali M. Y. Ashraf M. A. Mansoor S. Shahid A. A.. (2017). Knock-down of whitefly gut gene expression and mortality by orally delivered gut gene-specific dsRNAs. PLoSOne 12, e0168921. doi: 10.1371/journal.pone.0168921 PMC5207534 28045942

[B37] Wang Y. He Y. Liu S. Wang X. (2020). Mechanisms of plant virus transmission by the whitefly *Bemisia tabaci* . Chin. Sci. Bull. 65, 1463–1475. doi: 10.1360/TB-2019-0854

[B38] Wang Z. Z. Shi M. Huang Y. C. Wang X. W. Stanley D. Chen X. X. (2016). A peptidoglycan recognition protein acts in whitefly (Bemisia tabaci) immunity and involves in begomovirus acquisition. Sci. Rep. 6, 37806. doi: 10.1038/srep37806 27892529 PMC5124967

[B39] Yu Z. Wang L. Zhao J. Song H. Zhao C. Zhao W.. (2022). TOB1 attenuates IRF3-directed antiviral responses by recruiting HDAC8 to specifically suppress IFN-β expression. Commun. Biol. 5, 943. doi: 10.1038/s42003-022-03911-x 36085336 PMC9463440

