# Supplementary material for: Effect of silencing Bemisia tabaci TLR3 and TOB1 on fitness and begomovirus transmission
Source: Front Plant Sci. 2023 Mar 14;14:1136262. doi: 10.3389/fpls.2023.1136262 (PMC10043976; doi:10.3389/fpls.2023.1136262)
Supplement: Supplementary file 1 [file DataSheet_1.pdf]

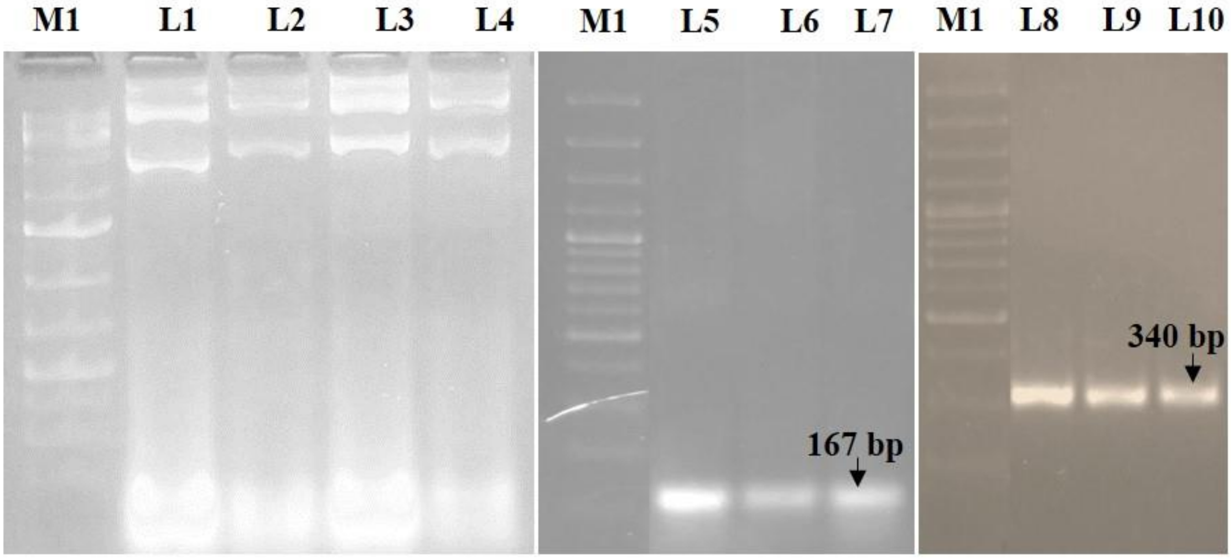

Supplementary figure 1. Synthesis of dsRNA targeting *B. tabaci TOB1* and *TLR3*. Total RNA isolated from recombinant *E. coli* HT115 cells (L1-L4, M1: 100 bp plus DNA ladder). Purified *TOB1* dsRNA (L5-L7) and *TLR3* dsRNA (L8-L10) purified from total RNA using DNase I and RNase A.
